# Supplementary material for: Exploring the Mechanism of the Intramolecular Diels–Alder Reaction of (2E,4Z,6Z)-2(allyloxy)cycloocta-2,4,6-trien-1-one Using Bonding Evolution Theory
Source: Molecules. 2023 Sep 22;28(19):6755. doi: 10.3390/molecules28196755 (PMC10574226; doi:10.3390/molecules28196755)
Supplement: Supplementary file 1 [file molecules-28-06755-s001.zip › molecules-2587280-supplementary.pdf]

Exploring the Mechanism of the Intramolecular Diels–Alder Reaction of (2*E*,4*Z*,6*Z*)-2(allyloxy)cycloocta-2,4,6-trien-1-one Using Bonding Evolution Theory

Abel Idrice Adjieufack [1,2,3,*], Jean Moto Ongagna [4], Jean Serge Essomba [2], Monique Bassomo Ewonkem [4], Mónica Oliva [5], Vicent Sixte Safont [5] and Juan Andrés [5,*]

1 Laboratory of Theoretical Chemistry (LCT), Namur Institute of Structured Matter (NISM), University of Namur, Rue de Bruxelles, 61, B-5000 Namur, Belgium
2 Physical and Theoretical Chemistry Laboratory, University of Yaoundé 1, Yaoundé P.O. Box 812, Cameroon; jeansergeessomba@gmail.com
3 Computational Chemistry Laboratory, High Teacher Training College, University of Yaoundé 1, Yaoundé P.O. Box 47, Cameroon
4 Department of Chemistry, Faculty of Sciences, University of Douala, Douala P.O. Box 2701, Cameroon; jean.monfils@yahoo.fr (J.M.O.); myewon@gmail.com (M.B.E.)
5 Analytical and Physical Chemistry Department, Jaume I University, Avda. Sos Baynat s/n, 12071 Castelló, Spain; oliva@uji.es (M.O.); safont@uji.es (V.S.S.)
* Correspondence: adjieufack21@gmail.com (A.I.A.); andres@uji.es (J.A.)

Content

1- Tables
2- Figures
3- Cartesian coordinates

Table S1-a. ΔG value (kcal/mol) evaluated in function of different XC DFT functionals as well as MP2 and CCSD(T) for the TSs of path-**a**. The basis set used in all cases was cc-pVTZ.

Species	B3LYP	M05-2X	ωB97X-D	M06-2X	B3LYP(D3)BJ	MP2	LC-BLYP	CCSD(T)
**TS1-a**	56.4	56.7	56.8	56.6	55.2	50.3	62.3	52.9
**TS2-a**	36.9	25.2	27.7	27.7	30.9	13.8	31.5	22.3

Table S1-b. ΔΔG value with respect the reference method (CCSD(T)) evaluated in function of different XC DFT functionals as well as MP2 for the TSs of path-**a**. The basis set used in all cases was cc-pVTZ.

Species	B3LYP	M05-2X	ωB97X-D	M06-2X	B3LYP(D3)BJ	MP2	LC-BLYP
**TS1-a**	3.5	3.8	3.9	3.7	2.3	-2.6	9.4
**TS2-a**	14.6	2.9	5.4	5.4	8.6	-8.5	9.2

Table S1-c. % deviation with respect the reference method in the ΔG values evaluated in function of different XC DFT functionals as well as MP2 for the TSs of path-**a**.

Species	B3LYP	M05-2X	ωB97X-D	M06-2X	B3LYP(D3)BJ	MP2	LC-BLYP
**TS1-a**	6.7	7.2	7.4	7.0	4.3	-4.9	15.1
**TS2-a**	65.5	13.0	24.2	24.2	38.6	-38.1	41.3
**Average**	36.1	10.1	15.8	15.6	21.5	-21.5	28.2

Table S1-d. M05-2X/cc-pVTZ electronic energies (E, Hartree·particle^{-1}), enthalpies (H, Hartree·particle^{-1}), entropies (S, cal·mol^{-1}·K^{-1}), and Gibbs free energies (G, Hartree·particle^{-1}) for the species involved in this intramolecular Diels-Alder reaction.

Species	E	H	S	G
**1**	-576.89732	-576.66064	135.571	-576.76179
**TS1-a**	-576.80443	-576.57180	133.679	-576.67153
**Int-a**	-576.89044	-576.65286	117.541	-576.74055
**TS2-a**	-576.86511	-576.63113	121.063	-576.72145
**2**	-576.93942	-576.70372	135.092	-576.80450
**TS1-b**	-576.85077	-576.61650	127.945	-576.71195
**Int-b**	-576.90371	-576.66739	138.582	-576.77078
**TS2-b1**	-576.83862	-576.60460	124.090	-576.69718
**TS2-b2**	-576.81278	-576.57855	120.915	-576.66876
**3-b1**	-576.93340	-576.69558	122.214	-576.78676
**3-b2**	-576.87073	-576.63266	118.334	-576.72095
**TS1-c1**	-576.80344	-576.56924	121.521	-576.65990
**TS1-c2**	-576.79208	-576.55790	123.864	-576.65031
**4-c1**	-576.91232	-576.67382	116.395	-576.76066
**4-c2**	-576.87645	-576.63857	121.205	-576.72899

Table S2. Topological parameters values of the electron density [$\rho(r)$] and its Laplacian [$\nabla^2\rho(r)$], $|V(r)|/G(r)$ ratio, and ellipticity (ε) at the C…C and O…C critical points in the TSs along the IMDA. In addition, instantly interaction energy(Eint), Lagrangian kinectic energy[G(r)], Energy density[H(r)], QTAIM Charge[Qe] and Eigenvalues of Hessian matrix[λ_{1-3}] at these previous BCPs(Bond critical points)

TS	BCP	$\rho(r)$ (a.u)	$\nabla^2\rho(r)$ (a.u)	$V(r)$ (a.u)	Eint	$G(r)$ (a.u)	$\|V(r)\|/G(r)$	$H(r)$ (a.u)	λ_1 (a.u)	λ_2 (a.u)	λ_3 (a.u)	Qe	ε (a.u)
**TS1-a**	**C2 ⋯ C7**	0.061	0.070	-0.044	0.022	0.031	1.433	-0.013	-0.059	-0.051	0.181	0.330	0.172
**TS2-a**	**C6 ⋯ C13**	0.054	0.043	-0.031	0.016	0.021	1.488	-0.010	-0.052	-0.047	0.142	0.367	0.106
	**C3 ⋯ C12**	0.053	0.049	-0.032	0.016	0.022	1.444	-0.010	-0.052	-0.043	0.144	0.359	0.208
**TS1-b**	**O10 ⋯ C11**	0.080	0.115	-0.069	0.035	0.049	1.413	-0.020	-0.092	-0.104	0.310	0.295	0.132
	**C3 ⋯ C13**	0.065	0.046	-0.039	0.020	0.025	1.542	-0.014	-0.067	-0.061	0.174	0.385	0.104
**TS2-b1**	**C4 ⋯ C12**	0.061	0.066	-0.041	0.020	0.029	1.421	-0.012	-0.064	-0.039	0.169	0.380	0.638
	**C7 ⋯ C11**	0.020	0.040	-0.034	0.017	0.022	1.553	-0.011	-0.060	-0.057	0.158	0.383	0.059
**TS2-b2**	**O10 ⋯ C12**	0.049	0.109	-0.091	0.046	0.059	1.540	-0.032	-0.132	-0.123	0.365	0.362	0.068
	**O9 ⋯ C11**	0.083	0.102	-0.070	0.035	0.048	1.468	-0.022	-0.113	-0.103	0.317	0.357	0.104
**TS1-c1**	**C4 ⋯ C12**	0.048	0.041	-0.027	0.014	0.019	1.460	-0.009	-0.045	-0.041	0.126	0.356	0.107
	**C7 ⋯ C13**	0.051	0.041	-0.029	0.014	0.019	1.474	-0.009	-0.049	-0.043	0.133	0.369	0.369
**TS1-c2**	**C2 ⋯ C12**	0.046	0.034	-0.024	0.012	0.016	1.482	-0.008	-0.042	-0.040	0.117	0.361	0.047
	**C5 ⋯ C13**	0.085	0.062	-0.060	0.030	0.038	1.589	-0.022	-0.078	-0.102	0.241	0.322	0.312

Table S3. Basin Populations (e), IRC coordinates (RX, amu$^{1/2}$ Bohr), C2–C7 bond lengths (Å), and energy (kcal/mol) with respect to the beginning of SSD-I along the **TS1-a** reaction pathway. For each SSD, the first value corresponds to the beginning of the domain while the second to its end. C and F mean "Fold and Cusp" catastrophes.

Basins	SSD-I		SSD-II		SSD-III	
**V(C2,C3)**	3.63	2.96	2.63	2.30	2.27	2.10
**V(C3,C4)**	2.12	2.99	3.03	3.24	3.26	3.37
**V(C4,C5)**	3.42	2.85	2.79	2.37	2.35	2.22
**V(C5,C6)**	2.12	2.75	2.84	3.22	3.24	3.37
**V(C6,C7)**	3.48	2.88	2.77	2.30	2.27	2.11
**V(C2)**	-	-	0.31	0.66	-	-
**V(C7)**	-	-	0.26	0.69	-	-
**V(C2,C7)**	-	-	-	-	1.44	1.92
Catastrophes	-	-	*F*	*F*	*C*	*C*
***d*(C2-C7)**	2.606	2.094	2.067	1.886	1.859	1.569
**E kcal/mol**	0.00	13.34	12.78	1.99	-0.36	-40.65
**Rx**	6.74	-0.32	-0.48	-1.60	-1.76	-15.72

Table S4. Basin Populations (e), IRC coordinates (RX, amu$^{1/2}$ Bohr), C3–C12/C6–C13 bond lengths (Å), and energy (kcal/mol) with respect to the beginning of SSD-I along the **TS2-a** reaction pathway. For each SSD, the first value corresponds to the beginning of the domain while the second to its end. C and F mean "Fold and Cusp" catastrophes.

Basins	SSD-I		SSD-II		SSD-III		SSD-IV		SSD-V	
**V(C3,C4)**	3.35	3.07	3.02	3.02	2.71	2.45	2.39	2.32	2.28	2.08
**V(C4,C5)**	2.25	2.72	2.98	2.98	2.91	3.17	3.22	3.26	3.29	3.40
**V(C5,C6)**	3.35	3.07	2.98	2.98	2.66	2.38	2.31	2.26	2.23	2.06
**V(C12,C13)**	3.42	3.29	2.84	2.84	2.72	2.38	2.31	2.23	2.16	1.94
**V(C3)**	-	-	-	-	0.29	0.51	0.56	0.61	-	-
**V(C6)**	-	-	-	-	0.26	0.51	-	-	-	-
**V(C12)**	-	-	0.21	0.21	0.27	0.46	0.52	0.57	-	-
**V(C13)**	-	-	0.25	0.25	0.33	0.56	-	-	-	-
**V(C3,C12)**	-	-	-	-	-	-	-	-	1.28	1.79
**V(C6,C13)**	-	-	-	-	-	-	1.20	1.31	1.41	1.83
Catastrophes			*FF*	*FF*	*FFFF*	*FFFF*	*CFF*	*CFF*	*CC*	*CC*
***d*(C3-C12)**	3.235	2.250	2.208	2.208	2.166	2.039	1.997	1.954	1.911	1.555
***d*(C6-C13)**	3.655	2.229	2.184	2.184	2.137	1.999	1.954	1.908	1.863	1.554
**E kcal/mol**	0.00	14.43	14.22	14.22	13.53	7.70	4.38	0.48	-3.85	-32.17
**Rx**	-10.47	0.00	0.30	0.30	0.60	1.50	1.79	2.09	2.40	6.86

Table S5. Basin Populations (e), IRC coordinates (RX, $amu^{1/2}$ Bohr), C3−C13 bond lengths (Å), and energy (kcal/mol) with respect to the beginning of SSD-I along the **TS1-b** reaction pathway. For each SSD, the first value corresponds to the beginning of the domain while the second to its end. C and F mean "Fold and Cusp" catastrophes.

**Basins**	**SSD-I**		**SSD-II**		**SSD-III**		**SSD-IV**	
**V(C2,C3)**	3.43	3.33	3.30	3.20	2.83	2.62	2.45	2.08
**V(C2,O10)**	1.47	1.57	1.61	1.85	1.93	2.07	2.19	2.43
**V(O10,C11)**	1.20	0.63	-	-	-	-	-	-
**V(C11,C12)**	2.05	2.24	2.29	2.88	3.00	3.12	3.18	3.45
**V(C12,C13)**	3.45	3.29	3.26	2.90	2.58	2.39	2.31	2.03
**V(O10)**	4.88	5.26	5.85	5.45	5.42	5.32	5.29	5.16
**V(C3)**	-	-	-	-	0.34	0.52	-	-
**V(C13)**	-	-	-	-	0.23	0.36	-	-
**V(C3,C13)**	-	-	-	-	-	-	1.03	1.84
Catastrophes					*FF*	*FF*	*C*	*C*
***d*(O10-C11)**	1.444	1.648	1.696	1.942	1.989	2.079	2.162	4.104
***d*(C3-C13)**	3.826	2.352	2.313	2.116	2.075	1.985	1.889	1.541
**E kcal/mol**	0.00	19.73	22.14	28.71	28.43	26.18	24.31	-4.48
**Rx**	-14.15	-1.89	-1.57	0.00	0.31	0.94	1.26	15.41

Table S6. Basin Populations (e), IRC coordinates (RX, $amu^{1/2}$ Bohr), C4−C12/C7−C11 bond lengths (Å), and energy (kcal/mol) with respect to the beginning of SSD-I along the **TS2-b1** reaction pathway. For each SSD, the first value corresponds to the beginning of the domain while the second to its end. C and F mean "Fold and Cusp" catastrophes.

**Basins**	**SSD-I**		**SSD-II**		**SSD-III**		**SSD-IV**		**SSD-V**		**SSD-VI**	
**V(C4,C5)**	3.46	3.17	3.13	3.13	3.09	3.09	2.67	2.42	2.37	2.37	2.32	2.10
**V(C5,C6)**	2.17	2.69	2.79	2.79	2.90	2.90	3.01	3.20	3.25	3.25	3.29	3.44
**V(C6,C7)**	3.47	3.15	2.78	2.78	2.67	2.67	2.56	2.33	2.28	2.28	2.24	2.04
**V(C11,C12)**	3.44	3.30	3.03	3.03	2.74	2.74	2.63	2.35	2.28	2.28	2.21	1.93
**V(C4)**	-	-	-	-	-	-	0.38	0.57	0.61	0.61	-	-
**V(C7)**	-	-	0.37	0.37	0.40	0.40	0.46	0.65	-	-	-	-
**V(C11)**	-	-	0.28	0.28	0.36	0.36	0.41	0.61	-	-	-	-
**V(C12)**	-	-	-	-	0.25	0.25	0.31	0.49	0.54	0.54	-	-
**V(C4,C12)**	-	-	-	-			-	-	-	-	1.24	1.84
**V(C7,C11)**	-	-	-	-			-	-	1.33	1.33	1.41	1.90
Catastrophes			*FF*	*FF*	*FFF*	*FFF*	*FFFF*	*FFFF*	*CFF*	*CFF*	*CC*	*CC*
***d*(C4-C12)**	3.382	2.203	2.170	2.170	2.137	2.137	2.103	2.002	1.968	1.968	1.933	1.544
***d*(C7-C11)**	4.360	2.216	2.180	2.180	2.144	2.144	2.108	2.002	1.967	1.967	1.932	1.555
**E kcal/mol**	0.00	43.80	44.04	44.04	43.78	43.78	42.96	36.78	33.55	33.55	29.86	-10.60
**Rx**	22.18	0.24	0.00	0.00	-0.24	-0.24	-0.48	-1.22	-1.33	-1.33	-1.71	-6.81

Table S7. Basin Populations (e), IRC coordinates (RX, amu$^{1/2}$ Bohr), O9–C11/O10–C11 bond lengths (Å), and energy (kcal/mol) with respect to the beginning of SSD-I along the **TS2-b2** reaction pathway. For each SSD, the first value corresponds to the beginning of the domain while the second to its end. C means " Cusp" catastrophes.

Basins	SSD-I		SSD-II		SSD-III	
V(C1,C2)	2.19	3.79	3.84	3.90	3.93	4.00
V(C1,O9)	2.43	1.71	1.64	1.60	1.55	1.46
V(C2,O10)	2.44	1.56	1.51	1.47	1.42	1.34
V(C11,C12)	3.45	2.23	2.19	2.16	2.12	2.02
V(O9)	5.25	5.76	5.82	5.88	5.13	4.92
V(O10)	5.24	5.85	5.31	5.29	5.23	5.04
V(O9,C11)					0.79	1.21
V(O10,C12)			0.64	0.75	0.85	1.22
Catastrophes	-	-	*C*	*C*	*CC*	*CC*
d(O10,C12)	3.722	1.706	1.664	1.623	1.582	1.433
d(O9-C11)	3.351	1.783	1.743	1.703	1.662	1.456
E kcal/mol	0.00	48.25	43.94	39.33	34.74	20.68
Rx	-18.22	1.21	1.52	1.82	2.13	4.22

Table S8. Basin Populations (e), IRC coordinates (RX, amu$^{1/2}$ Bohr), C4–C12/C7–C13 bond lengths (Å), and energy (kcal/mol) with respect to the beginning of SSD-I along the **TS1-c1** reaction pathway. For each SSD, the first value corresponds to the beginning of the domain while the second to its end. C and F mean "Fold and Cusp" catastrophes.

Basins	SSD-I		SSD-II		SSD-III		SSD-IV		SSD-V	
V(C4,C5)	3.34	3.08	3.03	3.03	2.70	2.28	2.23	2.23	2.20	2.01
V(C5,C6)	2.25	2.75	2.82	2.82	2.93	3.29	3.36	3.36	3.37	3.44
V(C6,C7)	3.44	3.12	3.08	3.08	2.71	2.26	2.25	2.25	2.20	2.06
V(C12,C13)	3.42	3.32	3.09	3.09	2.75	2.23	2.18	2.18	2.13	1.90
V(C4)	-	-	-	-	0.30	0.63	0.67	0.67	-	-
V(C7)	-	-	-	-	0.33	0.77	-	-	-	-
V(C12)	-	-	-	-	0.29	0.60	0.64	0.64	-	-
V(C13)	-	-	0.23	0.23	0.31	0.55	-	-	-	-
V(C4,C12)	-	-	-	-	-	-	-	-	1.38	1.91
V(C7,C13)	-	-	-	-	-	-	1.41	1.41	1.47	1.91
Catastrophes	-	-	*F*	*F*	*FFFF*	*FFFF*	*CFF*	*CFF*	*CC*	*CC*
d(C4-C12)	3.567	2.309	2.274	2.274	2.239	2.025	1.989	1.989	1.953	1.544
d(C7-C13)	3.730	2.271	2.233	2.233	2.196	1.970	1.933	1.933	1.896	1.545
E kcal/mol	0.00	28.67	28.46	28.46	27.82	12.34	8.00	8.00	3.39	
Rx	-14.50	0.00	0.25	0.25	0.51	2.04	2.29	2.29	2.54	9.40

Table S9. Basin Populations (e), IRC coordinates (RX, amu$^{1/2}$ Bohr), C2–C12/C5–C13 bond lengths (Å), and energy (kcal/mol) with respect to the beginning of SSD-I along the **TS1-c2** reaction pathway. For each SSD, the first value corresponds to the beginning of the domain while the second to its end. C and F mean "Fold and Cusp" catastrophes.

**Basins**	**SSD-I**		**SSD-II**		**SSD-III**		**SSD-IV**		**SSD-V**	
**V(C2,C3)**	3.60	3.14	2.74	2.74	2.64	2.46	2.39	2.25	2.24	2.11
**V(C3,C4)**	2.19	2.80	2.88	2.88	2.95	3.11	3.15	3.32	3.35	3.45
**V(C4,C5)**	3.38	3.10	3.07	3.07	2.72	2.53	2.45	2.26	2.22	1.99
**V(C12,C13)**	3.41	3.38	3.09	3.09	2.78	2.55	2.49	2.22	2.18	1.97
**V(C2)**	-	-	0.39	0.39	0.42	0.62	-	-	-	-
**V(C5)**	-	-	-	-	0.30	0.41	0.46	0.63	-	-
**V(C12)**	-	-	0.29	0.29	0.37	0.48	-	-	-	-
**V(C13)**	-	-	-	-	0.26	0.38	0.43	0.59	-	-
**V(C2,C12)**							1.25	1.57	1.63	1.89
**V(C5,C13)**									1.30	1.85
Catastrophes			*FF*	*FF*	*FFFF*	*FFFF*	*CFF*	*CFF*	*CC*	*CC*
***d*(C2-C12)**	2.975	2.203	1.992	1.992	1.957	1.889	1.854	1.722	1.693	1.518
***d*(C5-C13)**	4.222	2.378	2.345	2.345	2.313	2.248	2.217	2.081	2.044	1.572
**E kcal/mol**	0.00	52.74	52.95	52.95	52.72	50.75	48.96	37.47	33.98	0.03
**Rx**	-22.53	-0.24	0.00	0.00	0.24	0.72	0.96	1.92	2.16	7.89

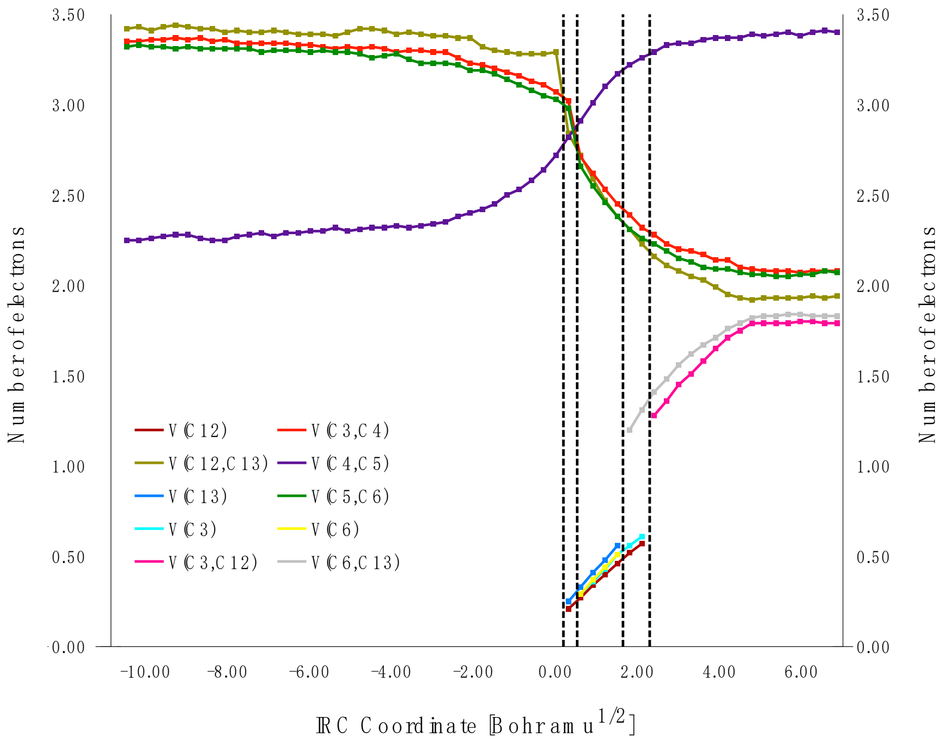

Figure S1. Population evolution (in e) of selected basins along the IRC associated to **TS2-a**

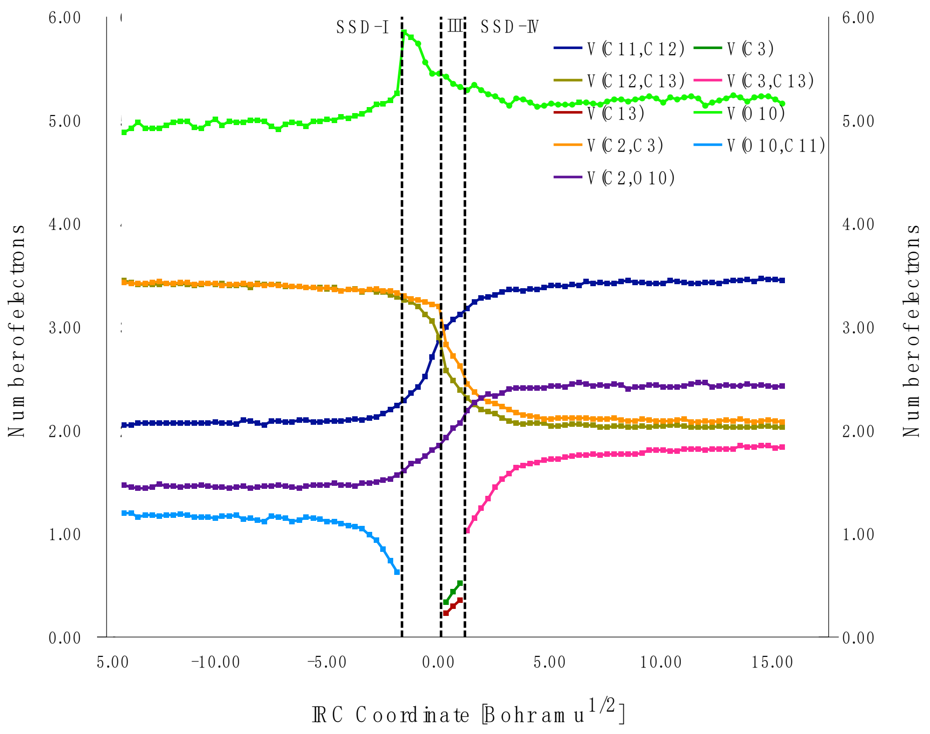

Figure S2. Population evolution (in e) of selected basins along the IRC associated to **TS1-b**

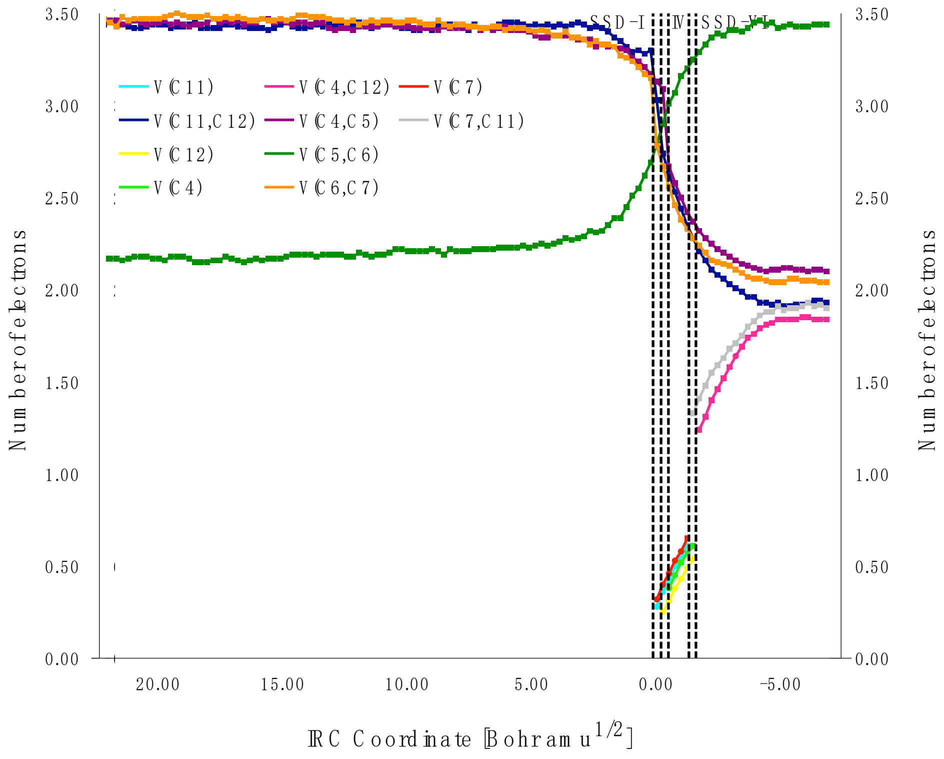

Figure S3. Population evolution (in e) of selected basins along the IRC associated to **TS2-b1**

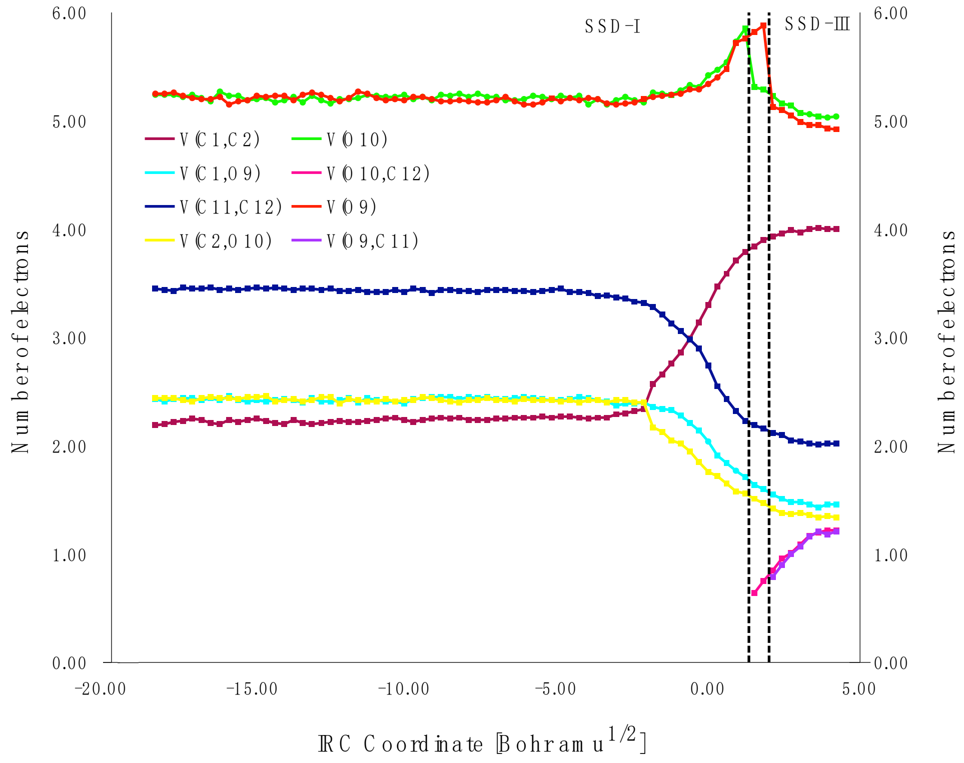

Figure S4. Population evolution (in e) of selected basins along the IRC associated to **TS2-b2**

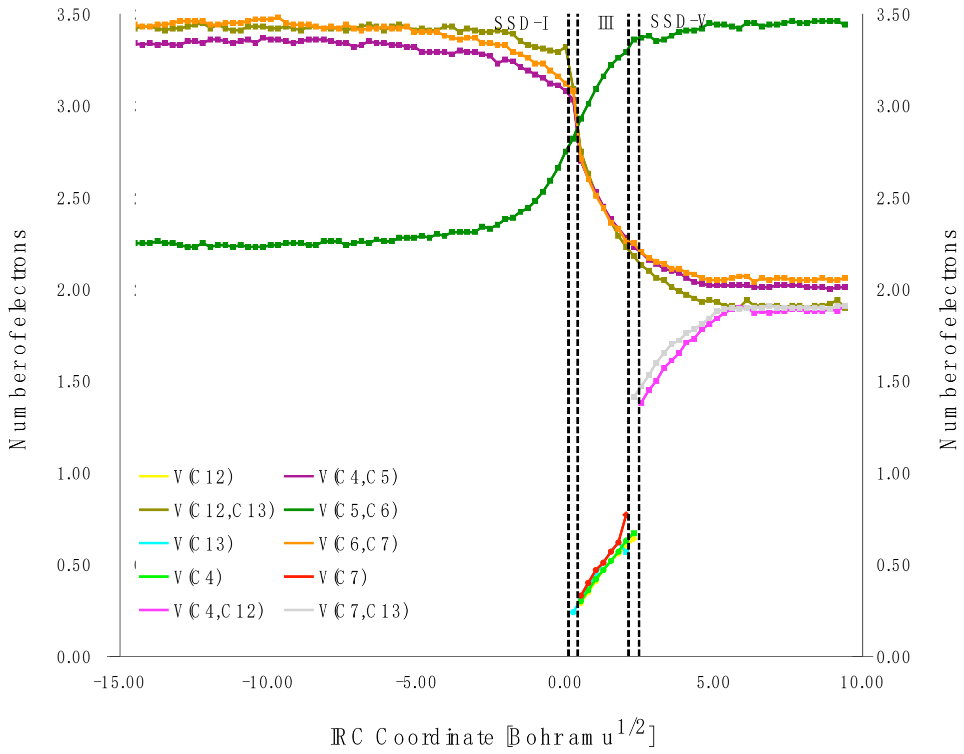

Figure S5. Population evolution (in e) of selected basins along the IRC associated to **TS1-c1**

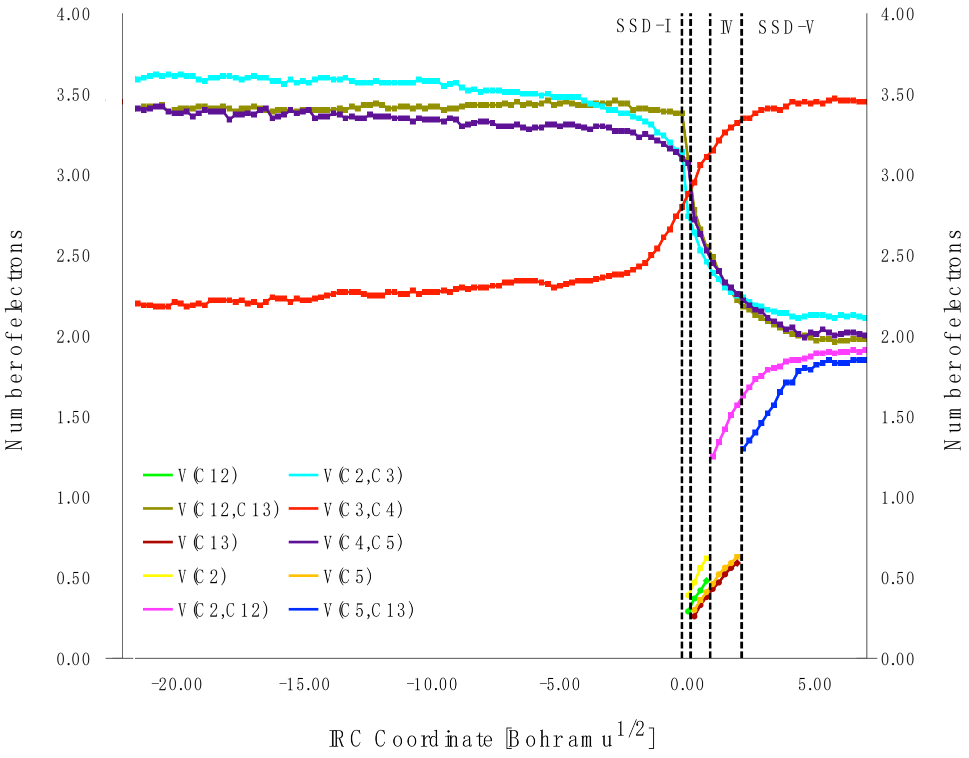

Figure S6. Population evolution (in e) of selected basins along the IRC associated to **TS1-c2**

Cartesian coordinates of the stationary points found

1

C	0.11582100	0.49620300	-1.16173800
C	-1.25976700	1.10392000	-1.20536200
C	-1.58666400	1.56776900	0.17529800
C	-2.32900100	0.85908600	1.02746200
C	-2.82824800	-0.46708300	0.75284100
C	0.25796100	-0.82486200	-0.49945600
C	-0.73000500	-1.63274600	-0.06525600
C	-2.14318800	-1.49203800	0.21098800
O	1.06814400	1.05204300	-1.66096400
O	1.49656500	-1.39759200	-0.55902700
C	2.60609300	-0.65456100	-0.00937800
C	2.27235300	-0.05842700	1.31764400
C	2.35295500	1.23960800	1.55713500
H	-1.98600700	0.36364200	-1.53357800
H	-1.21700600	1.93353600	-1.90290100
H	-1.22032900	2.54102100	0.46775500
H	-2.61971100	1.30806800	1.96756200
H	-3.83300200	-0.68346400	1.09025100
H	-0.36480900	-2.63131500	0.14458000
H	-2.68022400	-2.42864400	0.14296700
H	2.92203200	0.11093600	-0.70716300

H	3.38555900	-1.40426100	0.09321700
H	1.95140100	-0.74298600	2.09202100
H	2.64833900	1.92533900	0.77567600
H	2.12622300	1.65131000	2.52847300

TS1-a

C	-0.99261900	2.38344800	0.14928600
C	-0.62550500	1.88783900	-1.10339900
C	-1.46878500	-1.47180700	-0.09516800
C	-0.40662300	-0.50814300	-0.59964800
H	-0.61659400	2.62166800	-1.89892100
H	-1.20522100	3.44260600	0.18429600
C	-1.54066400	1.53161500	1.13520700
H	-2.51582000	1.79400900	1.53028500
C	-0.99336600	0.28692400	1.30242700
H	0.07660000	0.26754500	1.37842300
C	-1.66331100	-1.06656300	1.38045800
H	-1.17542000	-1.76342100	2.05681500
H	-2.72908500	-1.03801600	1.58982500
O	-2.16819300	-2.18282300	-0.75501600
C	-0.71582800	0.52857100	-1.47541000
H	-1.38932100	0.30463000	-2.29490000
O	0.84084600	-0.85774600	-0.20702200
C	1.89129800	0.07962700	-0.46954600
C	3.17721100	-0.57046700	-0.09542300
H	1.72649300	0.99300700	0.10478100
H	1.85567000	0.33740500	-1.52845600
C	4.06302400	-0.00828400	0.70771600
H	3.36601000	-1.53853500	-0.53829200
H	3.87481800	0.95543000	1.15895500
H	5.00165800	-0.48834200	0.93450000

TS2-a

C	0.88433500	-2.05409200	0.50231300
C	0.52683000	-1.20362000	1.54527900
C	-2.04024000	0.17483800	0.04012300
C	-0.53266400	0.48233100	-0.00327300
H	0.75892400	-1.47518700	2.56356700
H	1.44044100	-2.95562300	0.70840200
C	0.72815200	-1.59616700	-0.79030600
H	1.12968500	-2.16452800	-1.61548200
C	-0.41072800	-0.67522500	-1.05514700
H	-0.39123900	-0.25436500	-2.05511000
C	-1.83817300	-1.15496700	-0.67928200
H	-2.52685600	-1.35174300	-1.49374400
H	-1.83461500	-1.98880100	0.01848000
O	-2.98849300	0.79619200	0.39708300
C	0.13952800	0.09253500	1.26812700
H	0.00821000	0.79411800	2.08060800
O	-0.20735800	1.77315000	-0.40981000
C	1.12986600	2.13694900	-0.03506800

C	1.95580900	0.90768500	0.21877400
H	1.09656400	2.76365000	0.85385100
H	1.52070400	2.72621500	-0.86028100
C	2.25387700	0.02954100	-0.79686700
H	2.54497900	0.88541400	1.12113600
H	1.95340000	0.27254000	-1.80605200
H	3.08706000	-0.64527900	-0.70160100

TS1-b

C	0.18426400	1.51722100	0.26609500
C	1.68295800	1.45211400	0.40421300
C	2.15242200	0.76509300	-0.84077700
C	2.36247600	-0.54702500	-0.93065600
C	2.16448900	-1.49245900	0.15457200
C	-0.63675900	0.38512900	0.82316200
C	-0.21027200	-0.95047600	0.95783500
C	1.10746500	-1.59695300	0.97180800
O	-0.36051600	2.43608400	-0.29644100
O	-1.86867400	0.63404300	0.98072100
C	-2.73501900	0.15398200	-0.68971900
C	-2.39396100	-1.18458400	-0.71391600
C	-1.06298500	-1.53338100	-0.88917300
H	1.97072900	0.88726900	1.28653900
H	2.05923600	2.46862200	0.45168600
H	2.30552400	1.38942400	-1.70876700
H	2.76922100	-0.94360000	-1.85169900
H	2.94881300	-2.22633100	0.28666800
H	-0.93381100	-1.51078900	1.53085900
H	1.16337400	-2.39685300	1.69848700
H	-2.15869400	0.88037600	-1.24120700
H	-3.73354100	0.45751400	-0.42361700
H	-3.06800900	-1.90092100	-0.26781700
H	-0.42032200	-0.91092900	-1.49337200
H	-0.75947000	-2.56677400	-0.82266100

TS2-b1

C	-1.60534300	-0.56318400	0.14117600
C	-1.73291800	0.37816200	-1.01094600
C	-0.76544600	1.54533600	-0.89430000
C	-0.42919300	2.13783500	0.31463400
C	0.25752100	1.54495700	1.36551800
C	-0.36613600	-1.41902100	0.03106700
C	0.84294800	-1.06716600	0.84206200
C	0.97356900	0.35605100	1.38162500
O	-2.37634200	-0.67367800	1.05552900
O	-0.38711700	-2.34495900	-0.74184300
C	1.16980900	0.76182700	-1.52122600
C	2.03299600	0.36040000	-0.51208900
C	2.07943100	-1.08791200	-0.04956800
H	-2.74160100	0.78776600	-1.00587800

H	-1.57798400	-0.17699200	-1.93510600
H	-0.93603200	2.24528000	-1.70258000
H	-0.43271500	3.21966500	0.33958100
H	0.55477900	2.23324900	2.14463600
H	0.85052600	-1.78901700	1.66260500
H	1.72023600	0.34541000	2.16777200
H	1.38331700	1.68361100	-2.03601700
H	0.69896100	-0.00418200	-2.12185100
H	2.84800200	1.01230400	-0.24108000
H	2.97362600	-1.26956200	0.53896600
H	2.02312400	-1.82214600	-0.84824200

TS2-b2

C	0.37008600	1.03559500	-0.80355900
C	-1.02267600	1.58722900	-0.84515900
C	-1.61998300	1.47389300	0.52842700
C	-2.27018400	0.39580000	0.96006500
C	-2.50400500	-0.80304600	0.15519600
C	0.68609500	-0.30883400	-1.12848300
C	-0.10732800	-1.43353600	-0.52557100
C	-1.60570800	-1.51868700	-0.52206500
O	1.29009100	1.67903000	-0.23054800
O	1.92983700	-0.58478100	-1.02680700
C	2.04220800	0.60120500	1.19448000
C	1.91290000	-0.75392300	0.84004300
C	0.56045900	-1.40467300	0.94505700
H	-1.60781800	1.01666400	-1.56151200
H	-0.97958900	2.62727200	-1.15697500
H	-1.50222300	2.31932700	1.19053000
H	-2.72348300	0.42179100	1.94271400
H	-3.53204700	-1.14138800	0.11418100
H	0.27082800	-2.33917300	-0.98730100
H	-1.99733200	-2.35231300	-1.08901900
H	1.30501700	1.05442400	1.83714400
H	3.02544100	1.04022200	1.21651000
H	2.79771300	-1.36679600	0.84967600
H	-0.09123100	-0.83674300	1.60036500
H	0.62411000	-2.42341700	1.31194500

TS1-c1

C	1.44623700	1.44887600	1.12392800
C	1.23956100	0.13835400	1.49428900
C	-1.46505800	0.87224000	-1.01436400
C	-1.97568200	-0.09542000	0.04410300
H	1.96346900	-0.36296400	2.12084200
H	-1.49590900	0.34365600	-1.96881500
H	2.34276000	1.95237900	1.45867500
H	-2.21605300	1.65874900	-1.08451500
C	0.75336100	2.06860100	0.07642600
H	1.22921900	2.98776200	-0.24292200
C	-0.09938900	1.56069000	-0.90986800

H	-0.04260100	2.18987300	-1.79305700
O	-3.09384300	-0.06342000	0.47805500
C	-0.82637100	-0.90670900	0.52777900
O	-0.35576000	-1.79601400	-0.35272300
C	-0.18187200	-0.24212200	1.48653000
H	-0.81717300	0.45481900	2.01851900
C	1.04485400	-2.07831100	-0.22601500
H	1.23282900	-2.86397200	-0.95127100
H	1.24708100	-2.45822700	0.77388600
C	1.80710700	-0.82287100	-0.52727200
H	2.82258200	-0.75465300	-0.17722500
C	1.37471500	-0.03698600	-1.56779800
H	0.58744900	-0.40418500	-2.20686400
H	2.03839700	0.69419200	-1.99633700

TS1-c2

C	1.74791800	-1.17761000	0.38628000
C	0.83967400	-1.37272200	1.43131000
C	0.58774100	1.63946700	-0.87880500
C	-0.32641400	1.37578000	0.28520900
H	1.00611100	-2.26494200	2.01853100
H	0.22279100	1.13571100	-1.76998800
H	2.45210300	-1.99863200	0.32398700
H	0.55412400	2.71068900	-1.04721000
C	2.41145400	0.05309600	-0.08569800
H	3.48846400	-0.03287700	0.00208000
C	1.99182500	1.22000600	-0.56371000
H	2.74901600	1.96367800	-0.76170500
O	-0.67914200	2.27004900	1.00970200
C	-0.93528900	0.01890000	0.59949800
O	-2.30363300	0.06150600	0.27867700
C	-0.42059200	-0.81524300	1.57825100
H	-1.13020900	-1.30789900	2.22775200
C	-2.10994800	-0.37112700	-1.08812700
H	-1.90818100	0.48303800	-1.73190100
H	-3.00287500	-0.88332300	-1.43299700
C	-0.91917800	-1.29642500	-0.89609900
H	-1.25166200	-2.15510000	-0.32673000
C	0.32934200	-1.45897700	-1.46023700
H	0.76113800	-0.74218800	-2.13781200
H	0.74217800	-2.45147500	-1.51826400

Int-a

C	1.64218400	-1.26795600	-0.36695800
C	2.56664900	-0.19346500	0.18922600
C	1.33475600	0.38593700	0.94513400
C	1.16670900	1.86053300	0.89989500
C	0.60181400	2.43798600	-0.16069000
C	0.38749200	-0.46895700	0.02603300
C	-0.10608000	0.31189000	-1.14753400
C	0.02926700	1.63667000	-1.23522700

O	1.80315900	-2.35458400	-0.81969500
O	-0.58929600	-1.26196100	0.63079600
C	-1.75001000	-0.54486400	1.06525100
C	-2.89560100	-0.73963900	0.12518000
C	-3.62844000	0.25015200	-0.35468900
H	2.91427700	0.47373900	-0.59694000
H	3.40452700	-0.54469000	0.78071600
H	1.30102800	-0.00659500	1.95759900
H	1.52027800	2.45301700	1.73065700
H	0.52004800	3.51195000	-0.22407700
H	-0.60400400	-0.25701200	-1.91813000
H	-0.34999500	2.15084200	-2.10569100
H	-1.52417300	0.51577000	1.18049700
H	-2.00024900	-0.95359200	2.04252900
H	-3.11325500	-1.76675200	-0.13530200
H	-3.40181200	1.27851100	-0.10997200
H	-4.47001400	0.06745400	-1.00442200

Int-b

C	0.14756500	1.46159900	0.16414600
C	1.64563300	1.51947100	-0.00995900
C	1.99082900	0.64528700	-1.18830600
C	2.16037700	-0.67089300	-1.13210700
C	2.06926400	-1.48092800	0.08843800
C	-0.37557900	0.42000900	1.15723900
C	-0.37126800	-1.04014400	0.75973600
C	1.02731200	-1.60795800	0.90605700
O	-0.61592700	2.17439500	-0.42991000
O	-0.72943200	0.80978700	2.23800400
C	-3.09678900	0.00995500	-1.14863000
C	-2.48783200	-1.00815400	-0.56477800
C	-1.01144500	-1.25391700	-0.62412600
H	2.13709700	1.16049000	0.89147300
H	1.92144700	2.55033400	-0.20752800
H	2.07565000	1.14414300	-2.14214200
H	2.44559000	-1.19089200	-2.03753600
H	2.95600600	-2.05332700	0.33277300
H	-0.99406100	-1.52375000	1.50902600
H	1.16502000	-2.22767500	1.78064200
H	-2.54343300	0.73967000	-1.72074900
H	-4.16188500	0.15305200	-1.05432000
H	-3.06474500	-1.70811600	0.02732800
H	-0.55487300	-0.60280100	-1.36444900
H	-0.80733500	-2.28055500	-0.92553600

2

C	2.00802500	0.07992700	0.06333000
C	1.72169300	-1.18345500	-0.75274300
C	0.34191700	-0.57583300	-1.11239600
C	-0.97000400	-1.31646400	-0.81831900
C	-0.85280800	-1.99420300	0.51358600

C	0.55753800	0.53291900	-0.04945500
C	-0.38299800	0.25426200	1.14653800
C	-0.53366600	-1.18286200	1.51449500
O	2.99209200	0.57037500	0.52113700
O	0.28162900	1.85417700	-0.42398200
C	-1.10183200	2.11334800	-0.11760400
C	-1.64939200	0.79817800	0.42774900
C	-2.05110200	-0.20969800	-0.67715600
H	1.67122900	-2.06854700	-0.12382700
H	2.41392700	-1.34879300	-1.57193100
H	0.33468900	-0.14129300	-2.10818400
H	-1.23478000	-2.00737900	-1.61214500
H	-1.01118000	-3.05446900	0.63349900
H	-0.09880600	0.88843800	1.98081700
H	-0.40550000	-1.50996400	2.53386200
H	-1.60614300	2.44656800	-1.02079800
H	-1.14678500	2.90251500	0.63032600
H	-2.46483800	0.96535500	1.12340100
H	-2.16091000	0.30627900	-1.63020100
H	-3.00490100	-0.67184900	-0.44021800

3-b1

C	-1.54757800	0.46179400	-0.23054400
C	-0.87396900	1.78456700	-0.04419000
C	0.67004300	1.69616300	-0.07738100
C	1.21950600	1.19896600	1.22836300
C	1.54442300	-0.06875600	1.43754300
C	-1.12044600	-0.70800800	0.65341300
C	-0.09005500	-1.65369100	0.11807700
C	1.36442800	-1.09406800	0.37620100
O	-2.42028500	0.26708100	-1.03969900
O	-1.63851700	-0.82699200	1.73415100
C	1.22316300	0.90327100	-1.27791900
C	1.40171600	-0.61172700	-1.09844900
C	0.19388500	-1.52881800	-1.39354500
H	-1.18955300	2.18581100	0.92014200
H	-1.24007100	2.43562100	-0.83389300
H	1.00147600	2.72570700	-0.19983800
H	1.31560400	1.91956700	2.02842800
H	1.90255700	-0.38369900	2.40657500
H	-0.29072100	-2.63548300	0.53285300
H	2.02827600	-1.93909700	0.52675200
H	2.20580500	1.31897300	-1.48772900
H	0.60737200	1.09630600	-2.15732200
H	2.30266400	-0.90751900	-1.62588700
H	0.51801700	-2.48168900	-1.79955100
H	-0.60170300	-1.13337500	-2.01555100

3-b2

C	0.38854000	0.88375800	-0.82627500
C	-0.94288000	1.53684100	-0.93561500

C	-1.58805000	1.55251500	0.42284300
C	-2.26637000	0.51586900	0.90837700
C	-2.51562600	-0.72930900	0.17600000
C	0.66027000	-0.40435800	-1.06308600
C	-0.14182500	-1.52713300	-0.44093100
C	-1.64534300	-1.53143900	-0.43763800
O	1.34674300	1.60651400	-0.17963700
O	1.98394100	-0.74808900	-0.75734600
C	1.91306000	0.85387100	0.93042000
C	1.87806600	-0.66207500	0.66867800
C	0.54619700	-1.41061200	0.97170600
H	-1.55301500	0.97557100	-1.63761900
H	-0.81186200	2.54938900	-1.31118600
H	-1.48323200	2.44493500	1.02257000
H	-2.74974700	0.61518800	1.87184800
H	-3.55661200	-1.02680900	0.13272400
H	0.18383400	-2.45741700	-0.89587100
H	-2.07661400	-2.37667100	-0.95623900
H	1.36192200	1.12307400	1.83011100
H	2.94075800	1.18873700	1.01153600
H	2.73686000	-1.13709300	1.13226300
H	-0.08898600	-0.84428400	1.64604200
H	0.73498700	-2.38957800	1.40280500

4-c1

C	1.01041200	1.60764000	1.13257100
C	1.32761700	0.12625900	1.19208900
C	-1.45107000	0.57946400	-1.12476400
C	-1.88865300	-0.30778900	0.04499400
H	2.07115100	-0.08828200	1.95479700
H	-1.48819700	-0.02801600	-2.02942600
H	1.26921800	2.22419100	1.98106600
H	-2.21590000	1.34689800	-1.20697800
C	0.27803100	2.09381100	0.13561500
H	-0.04069600	3.12608200	0.15372100
C	-0.04297500	1.26358900	-1.08281200
H	-0.09877900	1.95670300	-1.92180300
O	-2.96744000	-0.22218100	0.56728700
C	-0.70434800	-1.07130200	0.50738800
O	-0.10347300	-1.80844800	-0.45367100
C	-0.05291600	-0.43865300	1.49011200
H	-0.66140300	0.10734500	2.19299800
C	1.31423300	-1.84638100	-0.29586200
H	1.69875100	-2.34187300	-1.18018600
H	1.56404700	-2.42342100	0.59570700
C	1.78188900	-0.40930200	-0.18309200
H	2.86700900	-0.37972500	-0.23188100
C	1.20099500	0.39235500	-1.36897700
H	0.99821000	-0.28380800	-2.19783700
H	1.96459700	1.09080100	-1.70268500

4-c2

C	1.57681400	-1.20133700	0.09614800
C	0.93603800	-1.08332300	1.48067700
C	0.58109500	1.61929600	-0.92427400
C	-0.46308500	1.32541000	0.13447800
H	1.55138900	-1.38959300	2.31367200
H	0.28271900	1.14501800	-1.85887800
H	2.26452400	-2.04146000	0.13415600
H	0.54396200	2.69395300	-1.06769700
C	2.41673700	0.04140100	-0.11311100
H	3.46210800	-0.04347000	0.14973400
C	1.98906700	1.22010500	-0.54392400
H	2.71724600	2.01401500	-0.62022300
O	-0.95031500	2.22146300	0.77252600
C	-0.94145800	-0.10350300	0.44298900
O	-2.37499800	-0.12062000	0.23477000
C	-0.25653800	-0.53199700	1.69298000
H	-0.67073100	-0.35250300	2.67224000
C	-2.14310500	-0.67341300	-1.09755700
H	-2.07804000	0.12719700	-1.83433200
H	-2.90679700	-1.38965500	-1.37722600
C	-0.80092200	-1.20957800	-0.58739000
H	-1.07379400	-2.09968200	-0.02526300
C	0.59366100	-1.43869900	-1.10754000
H	0.87742000	-0.79449400	-1.93171100
H	0.70267200	-2.46224400	-1.45368700
